# Supplementary material for: Spatial Variation in Stream-Water Bacterial Communities Across Sites Differing in Wild Chinese Giant Salamander Detection Frequency
Source: Biology (Basel). 2026 Sep 18;15(18):1647. doi: 10.3390/biology15181647 (PMC13604818; doi:10.3390/biology15181647)
Supplement: Supplementary file 1 [file biology-15-01647-s001.zip › Data Related to “Spatial Variation in Stream-Water Bacterial Communities across Sites Differing in Wild Chinese Giant Salamander Detection Frequency”/Microbial Data/report/src/hyperlinks/Methods_QIIME2_EN.pdf]

Method

The method is only used as a reference for publication purpose. Customers are responsible for the related risks of duplicate checking.

1 ExperimentalProcedure

1.1 Sample Quality Control

Please refer to QC report for methods of sample quality control.

1.2 Amplicon Generation

16S rRNA/18SrRNA/ITS genes of distinct regions (16SV4/16SV3/16SV3- V4/16SV4- V5, 18SV4/18SV9, ITS1/ITS2, ArcV4) were amplified used specific primer (e.g. 16SV4: 515F- 806R, 18SV4: 528F-706R, 18SV9: 1380F- 1510R, et. al) with the barcode. All PCR reactions were carried out with 15 μL of Phusion High - Fidelity PCR Master Mix; 0.2 μM of forward and reverse primers, and about 10 ng template DNA. Thermal cycling consisted of initial denaturation at 98℃ for 1 min, followed by 30 cycles of denaturation at 98℃ for 10 s, annealing at 50℃ for 30 s, and elongation at 72℃for 30 s and 72℃ for 5 min.

1.3 PCR Products Quantification and Qualification

The PCR products were purified using magnetic bead purification. Samples were mixed inequi density ratios based on the concentration of PCR products. After thorough mixing, the PCRproduct s were detected and target bands were recovered.For the PCR products generated by amplification , PCR product detection will be performed, and the detailed method can be found in the QC report.

1.4 Library Preparation and Sequencing

Sequencing libraries were generated and indexes were added. The library was checked with Qubit and real-time PCR for quantification and bioanalyzer for size distribution detection. Quantified libraries were pooled and sequenced on NovaSeq6000 or DNBSEQ-G99 platforms, according to effectivelibrary concentration and data amount required.

2 Bioinformatics Analysis Pipeline

2.1 Paired-end Reads Assembly and Quality Control

2.1.1 Data Split

Paired- end reads were assigned to samples based on their unique barcode and truncated by cutting off the barcode and primer sequence.

2.1.2 Sequence Assembly

Paired-end reads were merged using FLASH (V1.2. 1 1, http://ccb.jhu.edu/software/FLASH/) (Magoc T et al.,2011), a very fast and accurate analysistool, which was designed to merge paired-end reads when at least some of the reads overlap theread generated from the opposite end of the same DNA fragment, and the splicing sequences were called raw tags .

2.1.3 Data Filtration

Quality filtering on the raw tags were performed using the fastp (Version 0 .23 . 1) software to obtain high-quality Clean Tags (Bokulich NA et al.,2012).

2.1.4 Chimera Removal

The tags were compared with the reference database (Silva database (16S/ 18S), https://www.arb-silva.de/; Unite Database (ITS) , https://unite.ut.ee/) to detect chimera sequences, And the effective tags were obtained by removing the chimera sequences with the vsearch package (V2.16.0, https://github.com/torognes/vsearch) (Edgar RC et al.,2011).

For customer primer-based projects, chimeric sequences are by default detected and removed by alignment against the Silva annotation database (https://www.arb-silva.de/).

If the customer provides paired-end raw data, chimeras will be removed using the corresponding database based on the specified target region: the Silva database (https://www.arb-silva.de/) for 16S/18S, and the Unite database (https://unite.ut.ee/) for ITS.If single-end data is provided, chimera removal is determined by data quality:no chimera removal will be performed for pre- processed clean data, while chimeras will be removed using the corresponding database based on the target region for unprocessed raw data.

2.2 ASVs Denoise and Species Annotation

2.2.1 ASVs Denoise

For the Effective Tags obtained previously, denoise was performed with DADA2 or deblur module in the QIIME2 software to obtain initial ASVs (Amplicon Sequence Variants) (default: DADA2).

2.2.2 Species Annotation

Species annotation was performed using QIIME2 software. For 16S/18S, the annotation database is Silva Database, while for ITS, it is Unite Database. For unregular region, Unregular region: Micro_NT by default (a sub library obtained by extracting archaea, fungi, viruses, and bacteria from the NT) .

Note:

1. The fasta file (SILVA_138.1_SSURef_NR99_tax_silva.fasta) from the Silva offical website only provide species name rather than taxnonomic information, and thier taxonomic information file is incomplete. Therefore, we need use species name to get their taxonomic information from NCBI. At first, taxonomic information file from Silva offical is used to annotate, for those sequences that can not be annotated, a dmp file provided by NCBI would be use to supplement the taxonomic information.

2. Annotation principle of Micro_NT: perform sequence alignment (BLAST) between our sequence and sequence in Micro_NT. Then obtain the top20 result according to the score, screen by the maximum bit-score, use LCA algorithm to infer the least common ancestor. Due to the large amount of unclassified information in the NT, in order to ensure the accuracy of the data, the annotation effect is greatly reduced, resulting in more unclassified results. So when we use LCA algorithm to obtain species information, we will ignore unclassified species to reduce their impact on annotation.

2.2.3 Phylogenetic Relationship Construction

In order to study phylogenetic relationship of each ASV and the differences of the dominant species among different samples(groups), multiple sequence alignment was performed using QIIME2 software.

2.2.4 Data Normalization

The absolute abundance of ASVs was normalized using a standard of sequence number corresponding to the sample with the least sequences. Subsequent analysis of alpha diversity and beta diversity were all performed based on the output normalized data .

2.2.5 Relative abundance

Top 10 taxa of each samples at each taxonomic ranks(Phylum, Class, Order, Family, Genus, Species) were selected to plot the distribution histogram of relative abundance in Perl through SVG function.

2.2.6 Heatmap

The abundance information of top 35 taxa of each samples at each taxonomic ranks were used to drawn the heatmap, which visually display different abundance and taxa clustering. This was achieved in R through the pheatmap() function.

2.2.7 Ternary plot

Ternary plot for top 10 taxa at each taxonomic ranks can be used to show the abundance difference among three samples. It was performed in R with vcd() function.

2.2.8 Venn and Flower diagram

Venn and Flower diagrams visually display the common and unique information between different samples or groups. Venn and Flower diagrams were produced in R with VennDiagram() function and in perl with SVG function, respectively.

2.2.9 Phylogenetic tree

Phylogenetic tree, also called evolutionary tree, can describe the evolutionary relationship between different species. One hundred genera with the highest abundance in the samples were selected and performed sequence alignment to draw the phylogenetic tree in perl with SVG function.

2.3 Alpha Diversity

2.3.1 Alpha Diversity

In order to analyze the diversity, richness and uniformity of the communities in the sample, alpha diversity was calculated from 7 indices in QIIME2, including Observed_otus, Chao1, Shannon, Simpson, Dominance, Good's coverage and Pielou_e.

Three indices were selected to identify community richness:

Observed_otus – the number of observed species (http://scikit-bio.org/docs/latest/generated/skbio.diversity.alpha.observed_otus.html);

Chao – the Chao1 estimator (http://scikit-bio.org/docs/latest/generated/skbio.diversity.alpha.chao1.html);

Dominance – the Dominance index (http://scikit-bio.org/docs/latest/generated/skbio.diversity.alpha.dominance.html);

Two indices were used to identify community diversity:

Shannon – the Shannon index (http://scikit-bio.org/docs/latest/generated/skbio.diversity.alpha.shannon.html);

Simpson – the Simpson index (http://scikit-bio.org/docs/latest/generated/skbio.diversity.alph

a.simpson.html);

One indice was used to calculate sequencing depth:

Coverage – the Good's coverage (http://scikit-bio.org/docs/latest/generated/skbio.diversity.alpha.goods_coverage.html);

One indice was used to calculate species evenness:

Pielou_e – Pielou's evenness index (http://scikit-bio.org/docs/latest/generated/skbio.diversity.alpha.pielou_e.html).

2.3.2 Species accumulation boxplot

In order to evaluate the richness of microbial community and sample size. Species accumulation boxplot can be used to visualize, which performed with vegan package in R software.

2.4 Beta Diversity

2.4.1 Beta diversity

In order to evaluate the complexity of the community composition and compare the differences between samples(groups), beta diversity was calculated based on weighted and unweighted unifrac distances in QIIME2.

2.4.2 Beta diversity heatmap

Beta diversity analysis was used to evaluate differences of samples in species complexity. Beta diversity on both weighted and unweighted unifrac were calculated by QIIME software. Then a heatmap was created to display the unifrac distance between samples, which was realized in Perl.

2.4.3 Unweighted Pair-group Method with Arithmetic Mean (UPGMA)

A cluster tree is constructed on UPGMA, which is based on the weighted unifrac distance matrix. This is widely used in ecology for evolutionary classification. UPGMA diagram was drawn through the upgma.tre function within Qiime.

2.4.4 Cluster analysis

Cluster analysis was preceded by principal component analysis (PCA), which was applied

to reduce the dimension of the original variables using the ade4 package and ggplot2 package with R software(Version 4.0.3).

Principal Coordinate Analysis (PCoA) was performed to get principal coordinates and visualize from complex and multidimensional data. A distance matrix of weighted or unweighted unifrac among samples was obtained before transformation to a new set of orthogonal axes, by which the maximum variation factor is demonstrated by first principal coordinate, and the second maximum one by the second principal coordinate, and so on. PCoA analysis was displayed by ade4 package and ggplot2 package in R software(Version 4.0.3).

Non-metric multidimensional scaling (NMDS) was also implemented for data dimension reduction. Similar to the PCoA, NMDS also use the distance matrix but it emphasizes the numerical rank instead. The distance between sample points on the diagram can only reflect the

rank information rather than the numerical differences. NMDS analysis was implemented through R software with ade4 package and ggplot2 package.

2.5 Community difference analysis

A series of statistical analyses which inlcude Anosim, Adonis, Multi-response permutation procedure (MRPP), Simper, T-test, MetagenomeSeq and LEfSe, were performed to reveal the communitystructure differentiation.

Anosim, Adonis and MRPP analysis are non-parametric tests that analyze the difference between high-dimensional data group. They can test whether the differences between groups are significant greater than the differences within the group, which can determine whether the grouping is meaningful. All of them were performed with vegan package and ggplot2 package within R.

Simper can reveal the contribution of each species to the differentiation between groups. Top 10 species were selected and presented on the graph. It was performed in R with Vegan package and ggplot2 package.

MetagenomeSeq can showcase the species that display significant differences between groups. It was performed in R with metagenomeSeq package.

LEfSe is widely used to discover biomarkers and it can reveal metagenomic characteristics. To achieve this, a exclusive package named lefse was utilized.

2.6 Function prediction

PICRUSt (V1.1.4) is a package in R and is mainly used to predict the metagenomic functions based on marker genes. PICRUSt2 (V2.3.0) is the improved version of PICRUSt.

Tax4Fun (V0.3.1) is a R package that is widely used for intestinal and soil samples. In general, it can supply more accurate results when compared with PICRUSt, especially for soil samples.

BugBase is an excellent tool to discover the phenyotpe of microogranisms. It can classify the microbial communities according to seven phenotypes: Gram Positive, Gram Negative, Biofilm Forming, Pathogenic, Mobile Element Containing, Oxygen Utilization, including Aerobic, Anaerobic, and Cultivable Anaerobic, and Oxidative Stress Tolerance.

FunGuild is an excellent tool through python when working with fungi samples.

FAPROTAX through python can play a great role when elucidating the possible biochemical processes and elements in play.

2.7 Association analysis

To explore the symbiotic relationship between species and to reveal the environmental factor influence on community structures, 2D and 3D network diagrams were drew for visualization. Further analyses such as spearman correlation test, canonical correspondence analysis (CCA)/redundancy analysis (RDA) and dbRDA can be used to reflect the correlation between environmental factors and species abundance. All of these diagram and analysis were completed in R.

3 Reference

Bokulich NA, Subramanian S, Faith JJ, et al. Quality-filtering vastly improves diversity estimates from Illumina amplicon sequencing. Nature Methods. 2012;10(1):57-59. doi:10. 1038/nmeth.2276.

Edgar RC, Haas BJ, Clemente JC, Quince C, Knight R. UCHIME improves sensitivity and speed of chimera detection. Bioinformatics. 2011;27(16):2194-2200. doi:10. 1093/bioinformatics/btr381.

Magoc T, Salzberg SL. FLASH: fast length adjustment of short reads to improve genome assemblies. Bioinformatic. 2011; 27(21): 2957-2963. doi: 10. 1093/bioinformatics/btr507.

Wang Y, Guo H, Gao X, Wang J. The Intratumor Microbiota Signatures Associate with Subtype, Tumor Stage, and Survival Status of Esophageal Carcinoma. Frontiers in Oncology. 2021;11. doi:10.3389/fonc.2021.754788.
